# Recovery from Posttraumatic Stress Symptoms: A Qualitative Study of Attributions in Survivors of War

**DOI:** 10.1371/journal.pone.0070579

**Published:** 2013-08-07

**Authors:** Dean Ajdukovic, Dea Ajdukovic, Marija Bogic, Tanja Franciskovic, Gian Maria Galeazzi, Abdulah Kucukalic, Dusica Lecic-Tosevski, Matthias Schützwohl, Stefan Priebe

**Affiliations:** 1 Department of Psychology, Faculty of Humanities and Social Sciences, University of Zagreb, Zagreb, Croatia; 2 Unit for Psychological Medicine, Merkur Teaching Hospital, University of Zagreb, Zagreb, Croatia; 3 Unit for Social and Community Psychiatry, Queen Mary University of London, London, United Kingdom; 4 School of Medicine, University of Rijeka, Rijeka, Croatia; 5 East London National Health Service Foundation Trust, London, United Kingdom; 6 School of Medicine, University of Sarajevo, Sarajevo, Bosnia and Herzegovina; 7 University School of Medicine, Belgrade, Serbia; 8 Department of Psychiatry and Psychotherapy, Technische Universitat Dresden, Dresden, Germany; University of Sydney, Australia

## Abstract

**Objective:**

The study explored factors to which people traumatized by war attribute their recovery from posttraumatic symptoms and from war experiences.

**Methods:**

: In-depth interviews were conducted with two groups of participants with mental sequelae of the war in the former Yugoslavia: 26 people who had recovered from posttraumatic stress disorder (PTSD) and 17 people with ongoing symptoms of PTSD. Participants could attribute their recovery to any event, person or process in their life. The material was subjected to thematic analysis.

**Results:**

Eight themes covered all factors to which participants attributed their recovery. Six themes described healing factors relevant for both groups of participants: social attachment and support, various strategies of coping with symptoms, personality hardiness, mental health treatment, received material support, and normalization of everyday life. In addition to the common factors, recovered participants reported community involvement as healing, and recovered refugees identified also feeling safe after resolving their civil status as helpful. Unique to the recovered group was that they maintained reciprocal relations in social attachment and support, employed future-oriented coping and emphasised their resilient personality style.

**Conclusions:**

The reported factors of recovery are largely consistent with models of mental health protection, models of resilience and recommended interventions in the aftermath of massive trauma. Yet, they add the importance of a strong orientation towards the future, a reciprocity in receiving and giving social support and involvement in meaningful activities that ensure social recognition as a productive and valued individual. The findings can inform psychosocial interventions to facilitate recovery from posttraumatic symptoms of people affected by war and upheaval.

## Introduction

War and disaster related mental health consequences have been well documented, in particular posttraumatic stress disorder (PTSD) and depression [1]. Most research on correlates of war-related posttraumatic sequelae has focused on characteristics of the survivors (e.g. age, gender, education, employment status, prior experience with trauma, pre-traumatization mental health) [2], [3], [4], [5], [6], [7], [8], [9] and characteristics of the traumatizing experiences (e.g. severity, proximity, number of events experienced) [10], [11]. Such information can help to identify people at risk for developing severe mental health problems and for providing treatment and other forms of assistance to the survivors. War is the prototypical case of organized violence that challenges the basic human and moral categories, and questions the existential meaning of the self and others [12], [13]. Consequently, war-related trauma typically involves a complex set of interpretations of the reasons for the distress and the consequences for their health. People attribute their recovery - or lack of it - by constructing the meaning of events based on their understanding of the cause of their distress and on their perception of the environment [14], [15].

Mental health consequences of war can last several years after the ending of the conflict [16], [17]. The post-war environment is typically unstable, often unsafe and lacks supportive mechanisms to help recovery from posttraumatic symptoms. This applies to both forced migrants [18], [19] and people who stayed in the area of conflict [20]. Most people recover from trauma and, after some time, continue with their productive life [21]. Some receive professional medical care, but many recover without it. Some are able to rebuild parts of their former life, whilst others have to start from anew. In the aftermath of conflict, traumatized people remain active agents of their own recovery and use the available resources to the best of their capacities [22]. The ability to maintain good functioning after stress exposure appears more common than previously assumed [23]. Studying resilience is therefore important for a comprehensive understanding of human responses to stress and trauma. Such research should identify inherent and acquired biological and environmental characteristics which safe-guard mental health in the face of trauma [24], and consider the interphase of individual and environmental (social) factors [25].

Another theoretical model to interpret the attributions of recovery from trauma and subsequent stress is the Conservation of Resources theory - COR [26], [27]. It suggests that people strive to obtain, retain and protect their personal resources, either instrumental (e.g., money or shelter), social (e.g., social support or status) or psychological (e.g., self-esteem or sense of autonomy). The loss of resources is typical for people affected by war and uprooting. This includes the physical, social, and psychological demands of situations involving mass destruction and casualties, either because of pain, injury or devastation, or because of the symbolic and personal relevance of loss [28]. The loss of resources can diminish the capacity of individuals and communities not only to cope with a traumatic situation, but also to recover from its consequences. This is especially likely when individuals or communities have depleted psychosocial and economic resources due to forced relocation and socioeconomic disenfranchisement [27]. Furthermore, many trauma survivors struggle with a sense of injustice due to the way in which they have been exposed to traumatic events or treated during their aftermath (e.g., via discriminatory distribution of resources). Consequently, people and communities with strong personal or social resources are supposed to be in a better position to recover from traumatic experiences.

This study aimed to explore key resources and processes to which people traumatized by war attribute their recovery from posttraumatic symptoms and overcoming the war-related experiences psychologically healthy. This may help to understand why some people overcome initial symptoms and others do not, based on their own explanations and attributions. We conducted in-depth interviews with two groups of people affected by the war in the former Yugoslavia and now living in different countries and contexts: one group that had recovered from PTSD and another one that had not. The armed conflict in the former Yugoslavia in the 1990s resulted in large numbers of displaced people in the newly created states and of refugees in Western Europe. Prevalence rates of PTSD in community samples of those who experienced war and stayed in the region have ranged from 10.6% to 35.4% [29]. In refugees from this area settled in three West European countries [9] even higher rates of PTSD were found. The present study identified several factors of recovery processes, including specific factors that have not been reported before.

## Methods

### Ethics Statement

The study was approved by the relevant national ethics committees: Ethic Committee for Research with Human Subjects, Faculty of Humanities and Social Science, University of Zagreb; Ethics Committee, School of Medicine, University of Belgrade; Ethics Committee, University of Sarajevo Clinical Centre; Ethics Committee, School of Medicine, University of Rijeka; Ethics Committee, Technische Universität Dresden; Ethics Committee of Modena Municipality and Royal Free Hospital & Medical School Local Research Ethics Committee.

### Setting and Participants

The study was part of a large scale multi-centre community-based survey in eight countries [29]. It provided an unique opportunity to sample interviewees who were similar in socio-demographic characteristics and war experiences, but differed in the course of their recovery from posttraumatic symptoms. The rationale and methods of the larger study have been described in detail elsewhere [30]. The participants in the Balkan countries were randomly selected among those who had been directly exposed to war. Participants in Western European countries were identified from resident registers, ‘snowballing’ or through community organizations. Inclusion criteria were: born in former Yugoslavia, aged 18–65, experienced at least one war-related event, with the last event at or after the age of 16, and no mental impairment due to brain injury or organic cause. Participants reported on average 4.7 (SD 3.2) potentially traumatic experiences during the war [31], [32]. This included a range of events, from most reported “bombardment/shelling” (84.5%), followed by “lack of shelter” (64.5%), “being under siege” (40.1%), and murder or death of a close person due to violence” (35.9%), to witnessing murder or death, combat situation, serious injury, torture, kidnapping, concentration camp/prison incarceration and non-sexual and sexual assault by a known person. The distress at the time was rated for each type of experience on average between 3.60 and 3.97, i.e. all close to the maximum of 4 [31].

The participants for the current qualitative study were recruited in three Balkan countries, i.e. Bosnia and Herzegovina, Croatia, and Serbia, and three Western European countries, i.e. Italy, Germany, and United Kingdom. Based on the data of the epidemiological survey [29], [30] two sub-groups of participants were identified in purposive sampling. One sub-group included participants with persistent symptoms: positive diagnoses for current and past PTSD, as diagnosed by the structured mental health assessment interview (MINI) [33] and current IES-R [34] score of 22 or higher. The other group comprised participants with initial symptoms who had recovered since: negative diagnoses for current PTSD but positive for past PTSD, and current IES-R score equal or lower than 11. The sampling design was based on the purposeful heterogeneity model, with intended spread of gender and age in each sub-sample and participating centre as far as possible. They did not differ in exposure to war-related events from the main study. In each of the participating centres interviews with 2 to 4 participants with ongoing symptoms of PTSD (17 interviews) and 2 to 5 participants with past PTSD (26 interviews) were completed, resulting in 43 in-depth interviews. There were 23 male and 20 female interviewees, aged between 27 and 57 years. This number of completed interviews enabled theoretical saturation of data. As characteristic for qualitative studies, such purposive samples are not representative for the population, but represent experiences of people who had been directly exposed to war and either still lived in the affected areas or lived as refugees in three West European countries.

### Procedures and Measures

In the principal study face-to-face interviews were first conducted between January 2005 and November 2006. The data obtained in those interviews and used for the current study included demographic characteristics, potentially traumatic experiences before, during and after the war, and current PTSD status. Participants with PTSD were re-assessed during interviews using the same instruments about a year later. The time from exposure to war trauma was between 8 and 16 years, depending on each participant. All interviewers were trained in the assessments used in the survey [30]. The participants in the current qualitative study were interviewed for a third time about one year after the second assessment. At this time, the guide for conducting in-depth qualitative semi-structured interviews was used. The same interviewers who undertook the previous two interviews also conducted the in-depth interviews. Out of the 7 interviewers, 5 were qualified psychologists, one a sociologist and one an ethnologist. Five interviewers originated from the former Yugoslavia.

Participants’ age, gender, marital status, educational level, and employment status were initially obtained on a brief structured questionnaire. The history of potentially traumatic experiences was assessed using a specifically amended version of the Life Stressor Checklist-Revised (LSCL-R) [35]. It asks whether a participant had experienced any of 24 potentially traumatic events before, during and after the war. Current mental disorders were assessed using the MINI International Neuropsychiatric Interview [33], a structured diagnostic interview assessing the symptom criteria used in the Diagnostic and Statistical Manual of Mental Disorders IV [36] with published translations for the languages used in this study.

For the present qualitative study, a semi-structured interview guide was developed in consultation with all research centres and piloted on seven participants. Questions addressed personal experiences and not general opinions. After discussion in the multi-disciplinary research teams, the guide was modified and the procedure for interviewing agreed. The interview questions in the recovered group asked about the experiences and attributions of recovery to tease out if these were specific or also occurred in the group with persistent symptoms. The interview first addressed attributions of change in posttraumatic stress symptoms over time in terms of the reasons for the change or lack of change (*“In your opinion what were the reasons for changes in your symptoms of trauma? Was there anything specific that has happened to you and which you think caused these changes?”)*. Second, we asked about most helpful and unhelpful events or experiences over time *(“Over time what did you find most helpful to deal with your symptoms, and in what way has it helped you? What did you find unhelpful, and in what way?”*). Areas that were probed during the interview included changes in life circumstances (family circumstances, employment, housing situation, legal status – if refugee), informal and professional support received, and self-help.

The interviews lasted about 40 minutes and were conducted in the mother tongue of the interviewees, recorded and transcribed. The interviewees were modestly compensated for their effort. Written informed consent was obtained from all participants prior to each interview.

### Data Analysis

All interviews were transcribed by the interviewers in the participating centres, ensuring the removal of any identifying information to maintain anonymity. The first analyses included developing the coding frame, based on the analyses of 10 randomly selected interviews. Three analyst researchers independently proposed potential coding frames, and then met with the coordinating centre to discuss and agree on the optimal schedule. Appropriateness of the coding frame and inter-rater reliability were then tested on another 9 interviews which were independently coded by three researchers. The consistency of coding the contents into the themes was practically complete. In fact, the coders always agreed on the major theme to which the unit of analysis should be coded. In about 5% of cases they disagreed about coding the given part of the transcript into one of the sub-topics. These differences were discussed in detail and resolved. The resulting coding frame was used to code the rest of the transcripts line-by-line. In further work, when in doubt, the coding researchers first consulted each other, and, if required, the coordinating centre to resolve the issue.

The coded transcripts were next entered by the coordinating centre into the data file using the NVivo 7 [37] software program for the qualitative analysis. During this process, any inconsistency in initial coding was identified, available process notes consulted, discussed with the coordinating centre and reconciled. This process was supervised by the first author. The data file was used in further analyses using the thematic analysis approach [38], [39]. It allows for inclusion of a priori as well as emergent concepts during the process of indexing [40].

The second stage of the analysis involved the rearrangement of units of analysis (facets) based on the codes and their subsequent grouping into higher order conceptual topics and themes [40], [41]. They represent commonalities in the attributions of helpful and unhelpful experiences in both the group that had recovered from the symptoms of PTSD and the group with ongoing symptoms. Frequency counts of referenced units of analysis within each of the themes were also recorded.

The coding researchers were clinical psychologists trained in qualitative methodology. The thematic analysis and interpretation was led by the first two authors, one of whom has extensive experience with this approach.

## Results and Discussion

The analysis identified eight broad themes reflecting the factors which the participants considered to be helpful in their struggle with posttraumatic symptoms: 1) Social attachment and support, 2) Coping strategies, 3) Personality hardiness, 4) Mental health treatment, 5) Material support, 6) Normalization of everyday life, 7) Psychological safety, and 8) Community involvement. The number of participants in each group who listed contents referring to the identified themes is presented in Table 1.

**Table 1:** Factors identified as helpful in recovery from posttraumatic symptoms.

Theme/Factor of recovery	Number of participants making references to each theme/factor			
	Recovered group		Unrecovered group	
	n	%	n	%
1. Social attachment and support	25	96	14	82
2. Coping strategies	18	77	14	82
3. Personality hardiness	18	69	10	59
4. Mental health treatment	14	54	14	82
5. Received material support	14	55	6	35
6. Normalization of everyday life	13	50	4	23
7. Psychological safety	8	31	0	0
8. Community involvement	7	27	0	0
Number of interviewees	26		17	

There were neither clearly contradictory evidence nor inconsistencies in the analyzed reports. The only observable difference in factual events affecting the symptoms between the refugees living in the Western European countries and the people who stayed in the area of previous conflict, was that the refugees reported that they had to struggle to obtain residency permit or citizenship in the receiving countries. Such feeling of insecurity resulting from these facts is evident in some of the quotes: It was clearly related to maintenance of symptoms or their reduction once this source of insecurity was reduces. In the presented quotes participants who stayed in the area of conflict are coded as “S” and refugees as “R”.

The overview of findings is presented in Table 2. It shows eight identified factors of recovery from symptoms and specific topics constituting each factor. Most of the factors include more than one topic. More specific contents within each topic are presented as facets of recovery processes. Although in some cases these were the same for both groups, more subtle qualitative differences between the two groups were identified at this level. Six of the factors of recovery were relevant for both groups. However, even within the common factors, there were specific differences between recovered and unrecovered participants in attributing what was helpful for symptoms reduction. The commonalities and differences are documented and discussed below for each of the factors of recovery. These factors are a combination of factual events and processes in lives of the participants, and their cognitive interpretation of how these events affected their recovery from PTSD symptoms. Some of the participants used the phrase to “move on” with their life which reflects strength, positive attitude and hope, as one of core ingredients of recovery from trauma [42]. However, some participants saw broader psychological recovery from war experiences overlap with their recovery from the symptoms. The qualitative method enabled such insight into the subjective world of the participants.

**Table 2:** Factors to which recovery from war-related posttraumatic symptoms is attributed.

Factor of recovery	Topic	Facets of recovery process	
		Recovered group	Unrecovered group
Social attachmentand support	Family	Emotional bonding, patience, understanding for mental health problems	
		Attachment to children and responsibility for them	
		Behavioural self control to avoid harming family	
		Progress of children as asset for future life	Feeling well only when family members close by and available
		Family helps orientation to the future	Burden to family; worried about future
		Active role within family and reciprocalsocial transactions	Dependant on family, feeling a worthless member, passive role within family
	Friends	Opportunity to discuss war related experiences in a trusting and understanding environment	
		Emotional and instrumental support	
		Downward social comparison with friends who are worse off	
		Common experiences with veteran friends from the same unit	
		Other friends with corresponding interests	Primarily friends with same war-related experiences
		Making new friends	
	Informal network	Volunteers, boss at work, military commander, a priest, sport club members, neighbours	
		Being important to other people; other people show interest in them	
		Instrumental support	
	Professionals	Relations beyond strictly professional relationship important as reassurance and social acknowledgment	
Coping strategies	Active coping	Seeing tangible effects of own work	
		Having a paid job; productive family member	Prefer simple jobs that do not require much concentration
		Meaningful activities that ensure recognitionand reassurance of own value	Whatever activity to control thoughts and intrusive memories
		Feeling of self-efficacy, productive individual	Spending time off work with people to control intrusive memories
		Everyday work routine and job related responsibilities	
		Maintaining a paying job as an aspectof self-worth	
	Sharing traumaticexperiences	Important to get “those things out”	
		Avoiding people who constantly talk about war	
		Was relieving to share experiences, not practiced any more	Still relieving to share experiences
		Memory of suffering became integrated life experience	Hoping to forget the past or obsessively talking about the past
		Talking about war and losses not soimportant any more	Suppress intrusions by avoiding talking about these issues
			Self-taught redirecting thoughts to children and “cheerful topics”
	Openness to newlife experiences	Highly valued emotional and behavioural self-control	
		Able to continue working or interactingdespite agitation	Use of “time-out” to regain self-control when angry
		Able to deal with anger a constructive way	Attempt to self-control anger and recognize risk situations
		Renewed belief in people	Self-isolate to reduce irritation by other people
			Avoided by other people because of problems with emotional control
		Feeling at peace with past doings	Feeling “moral restlessness”
	Calming	Being alone in a quiet environment	
	Positive memoriesand hope	Memories related to time before war	
		Idealized sentiments about pre-war life	
		Hope for better future founded in own pre-war achievements	Symptoms increase after visiting homeland
		Pre-war memories strengthening and related to future plans	
Personality hardiness	Challenge	Self-attributes as: grit, defiance, spite, self-discipline, ability to enjoy a moment	
	Commitment	Self-efficacy, commitment to improve own situation, humour	Accepting things as they are feeling that nobody helped the recovery,
	Future outlook	Life optimism, future outlook, hope	Low expectancies about future,
Mental health treatment	Mental healthservices	Easy access to mental health services	
		Timely mental health interventions	
		Psychotherapy and medication both helpful	
	Psychotherapy	Opportunity for talking about problems and concerns with a professional	
		Clarity of the treatment structure	
		Therapy in the mother language	
		Unhappy with passive therapist	
		Awareness of recovery progress	Modest expectations from therapy but were aware of positive effects
		Compliance with therapy requirements	Embittered and chronically dissatisfied
	Psycho-education	Understanding of own psychological status	
		Understanding connection of mental health status and symptoms	
		Normalization of symptoms	
		Receiving practical suggestions what to do and not to do	
			Guidance from practitioners for managing provocative situations
	Medication	Medication as necessary part of healing	
		Unhappy with strong side effects	
			Medication helpful to clam when agitated
			Easy access to anxiolytics from GP and psychiatrists
			Anxiolytics often taken for hyper-arousal
	Relationship withprofessionals	Reassuring relationship with a practitioner	
		Trusting competencies of a practitioner	
		Trust in a practitioner generalized into trust towards other people	
Received material support	Housing	Reconstructing/building a house	
		Provision of accommodation from the authorities	
			Unhappy with poor accommodation
	Social benefits	Money allowance, food, clothing, schooling of children	
		Seen as temporary assistance to help get on own feet	Not seen as temporary
			Insufficient, low subsidy
Normalization of everyday life	Establishingeveryday routine	Children go to school, family together, living from own work, young family members have a perspective for schooling and work	
		Opportunity for employment and decent housing	
	Routine socialrelations	Feeling accepted in the local community	
		Enjoying normal changes in the family structure	
		Making new friends	
Psychological safety	Reducing lifeuncertainty	No references to physical safety	
		Obtaining citizenship status, work permit, accommodation	
Community involvement	Involved beyondnetwork of familyand friends	Volunteering	
		Modelling behaviour that can empower peers	
		Helping others to “pay debt” for the received help in the past	
		Strengthened by helping people who are more miserable	

### Social Attachment and Support

The most frequently mentioned recovery factor was social attachment and bonding to at least one person, and social support. The topics listed by both groups included family, friends, informal network and personal relations with professional care providers. In both groups families provided primary social attachment, and emotional and practical support. Emotional bonding, patience with the participants and understanding for their mental health problems were highlighted as helpful when struggling with PTSD symptoms. This is illustrated by the following statements:

*The family is most important in events like this… We got along well, mostly because of my wife, she had an understanding for me… but if she had criticized me, the situation* [with symptoms] *would have been worse when I went through my crisis.* (S 5575, recovered, male).

*All this would not have been enough if I did not have the help of my family, primarily my husband, who had enough patience and nerves to hold me by the hand, take me for walks … although I could hardly wait to come back home… but he had enough nerves to give me hope that I would get out of it all* (R 1321, unrecovered, female).

*I met my wife after the war, when I was feeling really bad, I started breaking down because of all that stress. She helped me a lot, somehow I calmed down and moved on. She still helps me a lot today*. (S 6174, recovered, male).

Children in the family had a special role in both groups. Participants reported feeling a mixture of attachment to children and responsibility for them. The children were a source of happiness and pride related to growing up and their successes, but also reassurance for the future. Their responsibility for wellbeing of children and seeing the success of own efforts reported as strong motives for the interviewees to recover or cope with ongoing symptoms.

*The care about the children motivated me to move ahead.* (R 4119, recovered, female).

*When I see that* (my daughter) *is the best in the school, it gives me strength to move ahead, to work, to take a job that I never thought that I would agree to. This gives me strength… every day.* (S 3209, unrecovered, female).

A responsibility towards family members was reflected in behavioural self control to avoid harming or shaming them (*I realized that my son was afraid of me, and he was still small. A few times he saw my outbursts and my reactions… What helped me? If it was not for my children, I would have no reason to live… I want them to have a normal life and never to go through what I went through – S 6074, recovered, male*). This helped to exercise better symptom control and was sometimes seen as contributing to recovery.

Family as a source of support had somewhat different meanings for the two groups. In the recovered group the family facilitated orientation to the future, mainly through the progress of children, which these participants understood as a part of the regular life cycle. The fact that they had children and grandchildren who were successful in school or held jobs, for example, was important for feeling secure for the future (*My son has finished college. With his diploma… life here will be easier, he married well…. I* (also) *have a wonderful son-in-law, my granddaughter is almost grown up… I see the family grow, with each year passing the granddaughter will be able to help us…* – R 1310, recovered, female). Some wanted a more active role within the family, as opposed to being overprotected by the family (*Sometimes I am bothered by (*questions like*)“What do you need mother?” I told them last evening: “Don’t pet me all the time, let me do some things by myself”*– S 7056, recovered, female).

In the unrecovered group, references were made about depending on family members, feeling worthless, and having a role of a passive family member (*Sometimes you feel that you are totally worthless, and then again you feel “let me just be here”, for the harmony in the family… I do not see much of my contributions nor usefulness to children or my wife, not even to myself* – R 1347, unrecovered, male). Managing symptoms was linked to family members being close by and available when needed, but there were no references to the distant future. It was clear that these participants were aware that their family carried a heavy load in supporting them because of the ongoing PTSD symptoms (*My wife very much understands me…. She precisely feels when I am not well… Then she says “Let us talk, you and me”… My wife supports me a lot* – S 5031, unrecovered, male). This was especially true regarding the spouses, and more often for women than men. Unrecovered females made very few references to receiving specific support from their families.

Friends were seen as a source of support in both groups. They provided opportunities to discuss war related experiences within a trusting and understanding environment. The network of friends was an important component of emotional and practical support for both groups. War related experiences were especially important topic for the veterans in both groups. Some of them felt that only the veterans with whom they served in the same military unit were a source of support, because they were the only ones who can understand it all (*We, who have been in the war can talk only among us about the things that happened in the war and understand each other. I feel best among the friends who were at the front line together with me… I can find a common language only with them…* – S 6010, unrecovered, male). Unrecovered participants reported that their primary network consisted only of people with similar experiences and that they could talk openly only with them. They felt better when comparing themselves with friends whose situation was worse than their own (*… if I learn about someone’s experience that was worse than what I went through, I may be a little upset, but I feel a relief that other people have fared worse than I have* – R 1078, recovered, male).

Recovered interviewees reported having had friends with similar interests, such as sports (*I met a few new friends* (as a refugee) *with whom I am still in contact. Through them I started playing volleyball… After a game we would sit down and talk… they helped me to start hoping again*… – R 2523, female, recovered). These friends provided practical support, spent time with them and played sports. They used to encourage them when they had difficult times and high level of symptoms in the past. References to new friends were rare among the unrecovered participants.

The supportive informal network included people like members of volunteer organizations who at some point in their life helped in a practical way, a senior colleague at work, a military commander, members of a sports club, neighbours who cared, a priest who was patient and brought food and clothing. The two groups did not differ in the contents of how informal networks supported them. The common key aspect was the feeling that they were important to other people and that other people showed an interest in them. It was important to be seen as “*any human being*” (*This woman, a hotel manager, helped to get proper medical help for my daughter, and took me to her friend who was a neuropsychiatrist… She made me feel that I was not alone, that my children will not remain alone* – S 7136, unrecovered, female). Getting practical support when needed was also reported as helpful, as it increased the feeling of being accepted and assurance.

Consistent with testimonies of being important to other people in the informal network, was the sense of being important as a person to various care providers. This included school staff (*My teacher (*of German*)… allowed me to call her at any time when I had an important problem … this was very important to me* – R 2523, recovered, female) and medical staff (psychiatrist, nurse, general practitioner, psychotherapist). An indicator of feeling important was having easy access to these care providers, e.g. being allowed to contact them outside of the regular working hours, and the flexibility of staff who bended formal rules in order to see the participants outside of the working hours or without proper paperwork (*My doctor will see me without an appointment and without formalities. This really helps* – S 6158, unrecovered, male). The feeling of being important was helpful in both groups.

Both groups assessed the received social support and interpersonal bonding as important for dealing with posttraumatic symptoms. However, there were differences between the two groups. Recovered participants were able to use this resource more, re-establish their intra-familial role better, and enlarge their informal network. Social attachment and support helped them to orient their life towards the future and gave them an active role in their family. They fully participated in the social life within the family, with friends and the informal network. Moreover, they were able to give and receive more or less equally in the social exchange. Consequently, they expected such support to continue in the years to come which was important to feel safe. In contrast, unrecovered participants were concerned that they were a burden to the family and were worried about the future of relations with important others. They were aware that they were mostly receiving social support and were not happy with that disbalance in their social relationships.

The importance of social support for recovery from trauma has been well documented across very different types of trauma [21], [43], [44], [45]. Studies show that the primary factor in recovery from adverse situations are relationships that provide care and support, acceptance, safety and trust, and offer encouragement, both within and outside the family. Receiving social support is usually considered sufficiently beneficial. However, this study highlighted the healing power of social support as a mutual exchange among the people in close relations. Differences in the meaning of social support identified between the two groups of participants in the present study imply that social support is a mutual process. Being only on the receiving end alone may not be sufficiently conducive to good mental health.

The study also revealed a unique aspect of social support provided by care professionals. The care providers who allowed informal relationships with the participants were considered exceptionally important sources of support, reassurance and acknowledgment. When this happened, the participants felt especially respected and valued as individuals. It is likely that this nurtured their need for social recognition, which can be affected during traumatic experiences and in the aftermath. This can be linked to the need for rebuilding self-esteem after trauma [46]. Similarly, comparing oneself with friends who had fared worse was considered helpful, which is a mechanism of increasing self-esteem through a downward social comparison [47].

### Coping Strategies

The second most frequently identified factor of recovery was effective coping with posttraumatic symptoms. Five strategies of coping were identified: activity, sharing traumatic experiences, openness to new life experiences, calming techniques, and positive memories and hope. Although the participants in both groups used these same coping processes to deal with the symptoms, there were qualitative differences between the two groups.

Data show that active coping was seen as most helpful for the healing or managing of symptoms in both groups. Recovered participants emphasized engagement in meaningful activities that ensured social recognition, improved their self-esteem and feeling of being a productive and valued individual as healing. Maintaining a paid job was as clear sign of self-worth (*It is not only the money, it is a simple proof that you are worth something* – R 1078, recovered, male). For the unrecovered group being active primarily meant doing whatever helped to keep thoughts of war-related experiences and intrusive memories under control (*… or I find something to do, like washing the car, and other things, mainly to do anything, trying not to think about that* – R 4027, unrecovered, male). They preferred simple jobs not requiring much concentration (*For the things that I do, no specific concentration is necessary* – S 6158, unrecovered, male). Socialising after work with other people was another way of keeping the disturbing memories away. Seeing practical effects of their work was important too (*Primarily (*working helps*) to create something that I can see as a result of (*at the end of*) the day* – R 1321, unrecovered, female).

Sharing traumatic experiences was another helpful coping strategy. The interviewees had learned that it was important to get “those things out”. Unrecovered participants still used this strategy, as opposed to the recovered group who had used it effectively in the past. Recovered participants reported that it had been relieving and symptom reducing to share their experiences, but that the memory of suffering gradually became part of their integrated life experience, and that they seldom talked about this anymore. Moreover, some of them avoided people who still talked about the war all the time. For some, neither talking nor thinking about the war and losses was important any more (*I almost completely forgot that, I do not want to talk about it, I live my normal life. I cannot change anything, the only thing I can do is upset myself if I keep thinking about it and coming back to it all the time… Simply, I forgot about it and continued to live a new life* – R 1123, recovered, female). As expected, intrusions of war-related memories were still present in the unrecovered group and some participants tried to suppress them by avoiding talking about these memories. They used self-taught techniques of redirecting thoughts to children and talking about “happy topics”. They hoped to forget what had happened to them. In contrast, some other unrecovered participants had the urge to talk about war experiences often, to whoever was willing to listen, justifying this by “not running away from what has happened“. As one of them said: *I am almost willing to pay a round of drinks (*to other people*) just to talk about it because (*my*) soul is full of this… I look for people to talk to them. To talk, explain…* – R 1347, unrecovered, male).

Awareness of new life experiences was reported by both groups. Among these, emotional and behavioural self-control was highly valued as helpful to manage symptoms. Recovered participants reported that they became successful in this, being able to continue working or interacting with people despite being agitated. They became able to accept their anger and cope with it in a constructive way (*I accepted that anger is OK, that it is normal to get angry and I learned to deal with it* – S 6074, recovered, male). Some recovered participants became aware that achieving “inner peace” and “moral peace with the past experiences” facilitated the recovery from symptoms (*After the war you have on your conscience if you should have behaved like that. I have clarified these things (*for me*) and I know that I could not have done differently* – S 6147, recovered, male). Another reported that it was most helpful when she became aware how hatred of the enemy would be destructive for her own life. This allowed her to let go past grievances and start recovering. Some reported a renewed belief in other people (*I believe now that not all the people are like I thought they were (*bad*), because I had really lost belief in them* – R 2523, recovered, female). Unrecovered participants still invested considerable efforts to manage strong emotions and hyper-arousal and felt embarrassed when losing emotional control. They typically used “time-out” and left the situation to regain self-control when they felt accumulating anger. It helped when they were able to recognize the signs of upcoming anger and agitation, but this was not always possible. Some became aware that other people started avoiding them because of their “temper” and potential loss of self control, which was embarrassing. When possible, they tried to avoid stressful situations and “provocations“. At the same time, they were aware that this may lead to self-isolation (*Other people are getting on my nerves* – S 6158, unrecovered, male). Unlike the recovered participants, the unrecovered group reported feeling “moral restlessness” (*I never felt sorry about what I had decided to do, because I had made my decision. But I have to live with my decision, my life is all screwed* – R 1348, unrecovered, male).

The calming behaviours were similar in both groups. Participants preferred to spend time alone in a quiet environment, such as a forest or park (*Only the nature does not get on my nerves* – S 615, unrecovered, male). Calming behaviours were more typically reported by men rather than by women.

Positive past memories were, as a rule, related to the time before the war. This was characteristic for both groups, whilst hopes for a better future were more common in the recovered group. Recovered participants used pre-war memories as a self-strengthening mechanism. They made more concrete references to good things that happened in their previous life, and related these experiences to expectancies and plans for the future. They appreciated having a new present life and used own achievements in the pre-war period as self-encouragements for the present *(… and then I say to myself, I do not have time for this and I remember only the things from the past that were nice* – R 2523, recovered, female). Unrecovered participants who reported positive past memories, in fact nurtured sentiments about the good pre-war life that had gone forever (*My life was left there*– S 5053, unrecovered, female). Some of them objected that their suffering from the war went unrecognized and that it would be helpful if it was thoroughly recorded, to show that this was a battle between the Good and the Evil. The refugee participants felt shaken and their symptoms went up each time they visited the area in which they had experienced the war. The observed differences between the two groups are consistent with failed coping mechanisms of depressed in contrast to resilient bereaved people [23].

### Personality Hardiness

Both groups identified the same personality characteristics as helpful to manage symptoms. They included confidence in own strengths and abilities, determination, self-efficacy and optimism. These remarkably correspond to the concept of personality hardiness [48], [49], [50] which is conceived as a broad personality style or generalized mode of functioning that includes factors of commitment (*I learned that I can influence (*different things*) and that this was my responsibility* – R 2523, recovered, female), control and challenge (*Some things a person has to carve out for himself. I managed to establish some (self) control* – S 4027, unrecovered, male).

The participants in the recovered group attributed their recovery from PTSD more directly to specific characteristics of their personality style. This included self-efficacy (*I did not come here to try, but to succeed* – R 3285, recovered, male), future outlook (*When I look into the future, I do it by defining goals… in the near future the main goal will be my family and me* – S 6147, recovered, male), hope and optimism (*I always looked for a bright side… I was (*never*) pessimistic… I felt that I was the one who had to organize our life in this small room… and to keep the good spirit of us all. Yes, my nature was my primary* (resource), *when everything seemed black, I found the light at the end of a tunnel* – R 1310, recovered, female), humour (*Humour, often at my own expense…. I was always my own best helper* – S 7113, recovered, female). Obviously these participants have used effective defence mechanisms to recover from posttraumatic symptoms.

Among the unrecovered group, specific references to personality style included accepting things as they are, having low expectancies about own mental health improvements (*I try to socialize with people… to force myself to behave as I should… I try to hope that things are going to be better, that I will overcome this situation… But I feel that I will always have these problems* – R 1347, unrecovered, male). They felt that nobody helped them recover (*… I told myself “You became grey because of this, calm down… you were forced to do that”. This means that nobody helped me (*to change such behaviours and recover*)* – R 1348, unrecovered male).

In sum, attributions to the role of personality in recovery from PTSD symptoms were broadly similar in the two groups. Whilst the same aspects of resilience were perceived as helpful, recovered participants were more able to link this with a perspective for the future. The only remarkable difference was that optimism was referred to by the recovered, but not unrecovered participants.

### Mental Health Treatment

In both groups participants referred to the received mental health assistance as helpful. Yet, this was more frequent in the recovered group than in the unrecovered one. Some reported that they used only medication (7), others referred only to psychotherapy (5), but most (16) mentioned receiving both medication and psychological therapy.

Both groups considered the following to be helpful in managing symptoms: therapy sessions during which they had opportunity to talk about own problems, worries and concerns; psycho-education during which they received practical suggestions as to what to do and not to do, better understanding of their psychological state and its connection with the symptoms, guidance about taking one step at a time towards improvement.

*When I feel really bad, I go to my doctor* (psychiatrist) *and talk to her. She was very helpful because she helped me understand what was going on with me… what bothers me. I cannot always recognize some of these issues, while she has experience with other veterans… and helps me understand what is going on with me.* (S 6158, unrecovered, male).

It was also a relief to have the own problems explained as ‘normal’. Trust in the competence of mental health professionals and their visible commitment to help were very important. The interviewees appreciated clarity of the treatment, especially structured procedures such as cognitive-behavioural therapy. They all believed that medication and therapy were both helpful for dealing with symptoms (*Most helpful was talking with them (*mental health professionals*) and a friendly advice how to help myself… Group therapies helped me reduce medication… Now I still use L, Z and R. (*brand names for two benzodiazepines and one atypical antipsychotic*)* – S 7136, unrecovered, female). Several refugees emphasized that talking with a bilingual therapist in their mother language was most helpful.

In the recovered group almost an equal number of participants had been on medication (4) or in therapy (4) or both (6). They considered specific contextual aspects to be helpful for symptom management in addition to medication. These included treatment compliance, receiving timely mental health intervention (i.e. opportunity to discuss symptoms with a mental health professional at the time when these were of primary concern to the participants). Easy access to health services was also seen as very helpful. Being able to monitor the own recovery progress was beneficial, and the treatment techniques that enabled this were considered superior to approaches that did not provide such procedures. A reduction of the prescribed medication and a consequent decrease in distressing side effects were considered an indicator of healing.

*The treatment has helped me and when I come to the treatment centre nowadays, I feel good. I am not ashamed any more to go there… I did not want to take the pills, because if I take two of them, they will kill me and I will not be able even to walk, or I will walk like a drunken person…. If I did not take anything, I would not be able to calm down. Then the doctor starting lowering the doze and it was better… I started going into these groups… Individual therapy also helped me.* (S 6074, recovered, male).

Specific references to treatment in the unrecovered group were mostly related to medication. Out of 14 participants 13 were still taking “pills”, some felt that this was constantly in “large doses”. Ten of the participants at some time in the past had been in psychotherapy (individual or group) and still took medication. Only one participant had experience with therapy only, but never with medication. For three participants medication was the only mental health intervention. The participants in this group felt that medication was helping them calm down when agitated and feeling explosive, but were very unhappy with strong side effects which made them dizzy, disoriented and low energy. They explained that it was very easy to get “pills” from their general practitioners and psychiatrists – they just had to ask for them (*I would go to the doctor and ask him to give me something to calm down, and I would get 2 mg or 5 mg of A. (*brand name for a benzodiazepine*)* – S 7160. unrecovered, male). None of the participants in this group mentioned that their doctors worked on reducing the medication. But many felt that their practitioners provided good advice about managing provocative situations.

One of the unrecovered interviewees was very unhappy with the therapist who was perceived as extremely passive during therapy. This left the patient with the feeling that the therapist was emotionally distanced, rejecting, and not supportive. In this case she was not able to see the structure of therapy, where it was leading and felt that there was no progress at all.

*I started going to counselling, but this was not helpful. She never asked me a single question, she did not* (help me to) *open up… This was a loss of time. I was thinking all the time “Well, God, why have they sent me to this woman? What do I need to do?” Because she was sitting there, quietly, not asking anything. She just said “Come on, tell me about yourself”. And I came from the war, I was afraid! I was afraid of everything… And what should I tell her? Who is this* (individual) *to me? If she is a counsellor, a psychiatrist, she should encourage you, not irritate you…. This was going on each week for a long time, just waste of two hours. So we sat there and were quiet … Or I talked about anything… Then I would start crying. I do not remember any more what the reason for crying was. Was this because this irritating woman was sitting across me and staring at me or? … Mostly I cried because of this. I could not be rude and tell her “Go to…”* – R 1363, unrecovered, female).

Dissatisfaction with mental health care was more often raised in the unrecovered group. Some patients had perceived positive effects despite modest initial expectations.

*I hoped that* (medical help) *would help me. My expectations were not excessive, I went into the treatment very honestly… I believed these people* (doctors) *and did not conceal anything, we spoke openly about everything, about my personal problems, what bothers me most… I think that all these therapies had* (somewhat) *positive effects.* (R 1347, unrecovered, male).

Particularly low satisfaction with mental health services was reported by interviewees who were very bitter, who felt that their suffering was senseless, and who reported failure to establish and maintain close relationships with other people. In one dramatic case, a participant complained less about the specific clinical symptoms and made ironic comments about psychotherapy:

*The war has destroyed my generation… You can go to counselling… This fat woman can listen to you and nod her head the whole day, but there is no use of it… They can try to, like, fix one individual, but this does not mean that they have fixed hundreds… of people who have lived through this… I despise the whole Bosnian society… This country was built on the blood and flesh of our fighters… And then it all fell into water… For the last seven years I live in the West and I do not see that there is much soul here…* (My) *country never tried to help us as* (honest) *people…* (R 1348, unrecovered, male).

Among the unrecovered group there was more scepticism about the treatment outcomes regarding symptoms. They considered medication as necessary, but it was either not liked because of side effects or taken only to control hyper-arousal. Some of the unrecovered participants fitted well the description of a proposed “Post-traumatic embitterment disorder” [51], [52].

### Material Support

For participants in both groups the most helpful material support was housing since this meant satisfying one of their basic needs. This included help with reconstructing or building a house, and being provided accommodation by the authorities (*Of course, when they (*the authorities) *started rebuilding homes, we knew that sooner or later we will also get the keys. This was relieving* – S 4119, recovered, female). Secondly, social benefits were mentioned and included money allowance, food and clothing, and help with schooling of children.

In contrast, unrecovered participants mostly considered this help as insufficient, and not necessarily temporary. They were unhappy with the poor accommodation and low subsidy that they were getting to cover the costs of living (*The (*subsidy*) for rent is small. What kind of help is this if I have to pay 8–9 thousand euro per year, and I get back at the end of the year only 1.000 euro* – R 3209, unrecovered, male). Overall, they felt that they should be helped more with monetary and material provisions.

The two groups differed only a little in how they perceived the role of material support in their lives. Some recovered participant saw this primarily as a temporary assistance that helped them to get on their own feet. It is important to note that the participants who had received any kind of material support, acknowledged that this improved their living conditions, but did not connect it with the recovery from trauma symptoms.

### Normalization of Everyday Life

Normalization of everyday life was a rather broad theme attributing healing from PTSD to the possibility to develop an everyday routine, perform standard social roles, attend school (*This started to look like normal life, children went to school…* – R 1347, unrecovered, male) or hold a job (*We started going to work. This was good because things started to become normal* – S 4508. recovered, female), fulfil responsibilities, and be confident about the future as a consequence of all this. Both groups agreed that this was an important factor in dealing with symptoms. However, it was twice as often raised by recovered than by unrecovered participants. In addition, recovered participants felt accepted in the local community, were able to enjoy the normal changes in the family structure, and enlarged their social network with new friends, which was consistent with the notion of connectedness, one of the core elements of recovery from trauma proposed by Hobfoll and colleagues [42].

### Psychological Safety

Feeling safe was reported as a component of recovery only by recovered participants (8), with equal number of males and females. During the interview a few unrecovered participants (6) described how they were helped into safety during the war, but made no references to feeling safe at the time of the interview and did not link this experience with a recovery from symptoms.

Physical safety in the current life was not an issue, while the psychological dimension of feeling safe was important primarily for refugees (*When I first got the residence permit, I felt that this affected my health and psychological (*wellbeing*)… this was the major contribution (*to my recovery*)* – R 2523, recovered, female). It was related to uncertainties regarding the residency status and (in)ability to plan the future life. Resolving this issue had positive effects not only on recovery from posttraumatic symptoms, but also allowed other healing factors, such as active coping and normalization of everyday life to come to the forefront.

### Community Involvement

Only recovered participants (7, of which six were women) reported that they were actively involved in the community beyond the immediate social network of family and friends (*I work on helping veterans. I want to help them resolve their problems in a more successful way than me back in 1996. They need help and through this I also help myself* – S 6147, recovered, male). They described volunteering and helping other people. Some felt that they were “paying the debt” that they had because other people had helped them in the past. Other people felt that they should serve as a role model that can empower their peers. Some of the participants derived strength from helping other people who were more miserable than they (*… that even I was able to help them* – R 2523, recovered, female).

Community involvement basically reflected strong altruistic motives, such as helping other people and being involved in humanitarian activities. This is consistent with the reciprocal altruism [53] as one of the powerful social norms that people derive self-esteem from.

### Comparison with Literature

The findings of this qualitative study are consistent with the model of mental health protection [5] that distinguishes three sets of protective factors: (1) personal attributes, including outgoing, bright, and positive self-concepts; (2) the family, such as having close bonds with at least one family member; and (3) the community that can provide assistance. Our findings also fit with the mental health protective system model [24]. It includes the subject-related factors and experiences, which in our case are social attachment and support specific to posttraumatic distress, coping strategies, hardiness personality, normalization of everyday life, feeling of safety and community involvement. The other set of resources in this model are targeted assistance from the society, with respect to our findings these included mental health treatment and material support. It seems that subject-related resources were differently available to the recovered and non-recovered participants. Such differences were most obvious in attributions of how helpful social attachment and support, coping strategies and personal hardiness were in the recovery. Received mental health treatment and material help, as targeted assistance from the society, were not perceived as differently helpful between the two groups.

The findings in the present study show that resilience factors may become effective and available at any time during the process of recovery from posttraumatic symptoms. They result from individuals’ ability to interact with their environments and use available resources [54]. Such processes not only protect against the overwhelming influence of risk factors [50], but are also conducive to recovery. We identified a range of factors that, in the view of our participants, had contributed to their recovery from lasting and clinically significant symptoms of exposure to trauma.

These processes include support by the family and social network, individual coping strategies, personal hardiness style as well as the professional mental health treatment. It seems that in the recovered group there was a cumulative effect of such factors that facilitated healing. They also fit with the definition of resilience [55] which refers to the capacity of individuals to navigate their way to psychological, social, cultural, and physical resources that may sustain their well-being and capacity to negotiate for these resources to be provided. Our findings also correspond to the principles of intervention and prevention following mass violence [56]. For example, social attachment and support in our study corresponds to connectedness in these principles; active coping and community involvement in our study are similar to sense of self- and community efficacy principle; personality hardiness style in our study includes the principle of promoting hope; psychological safety in the present study partially corresponds to the sense of safety element; one of the coping strategies identified in the present study is the same as the principle of calming. It is noteworthy that the same principles of prevention efforts soon after traumatic exposure were identified by our participants as healing factors over the course of many years after trauma exposure. However, the current study identified also other factors to which people attribute their healing from posttraumatic symptoms over time, such as the normalization of everyday life, and received mental health treatment. Reciprocity in receiving and giving social support, strong orientation towards the future, and participating in meaningful activities that ensure social recognition as a productive individual clearly distinguished the recovered and unrecovered participants.

### Strengths and Limitations

Unique to this study was the approach to include both people who had recovered from PTSD and those who still had ongoing clinically relevant symptoms. Both groups were asked to attribute the changes in their mental health status and posttraumatic symptoms to whatever factors they considered to be helpful of unhelpful. The sample was large enough for the qualitative study to achieve theoretical saturation. The participants were all affected by the wars in the former Yugoslavia, but had very different backgrounds and life histories, and were interviewed in different countries by trained interviewers. Such heterogeneity of the sample design allowed for a variety of experiences to be included in the analysis. Each step of data processing was rigorously monitored to ensure dependability and trustworthiness of data, which are equivalents of reliability and validity [57] in qualitative research. This was ensured through thorough examination of raw data, data reduction products and process notes [58].

The study limitations are that the participants were interviewed a year after their PTSD status had been assessed and symptoms may have changed in the meantime. It is also possible that the recovery or lack of it at the time of PTSD status assessment may have been temporary. However, the assessment procedure was based on the structured mental health assessment interview (MINI) which uses a precise scoring schedule for presence or absence of symptoms used to diagnose current and past PTSD. In addition, IES-R score was used in the standard way to differentiate the participants who reported high or low distress scores. Moreover, the interview material, specially the parts in which the participants referred to their symptoms, rather consistently shows that the change in the mental health status was not likely to have happened to the degree that might have affected the results.

### Conclusions

The qualitative thematic analysis yielded eight factors to which people attribute their recovery from war-related posttraumatic symptoms. Six of them were identified as healing by both participants who had recovered from PTSD and participants with ongoing posttraumatic symptoms. These included social attachment and support, various strategies of coping with symptoms, personality hardiness, received mental health treatment, access to material support, and normalization of everyday life. While these factors were healing for participants in both groups, there were specific qualitative differences between the two groups: The recovered participants reported having reciprocal social relations, proactive coping styles, and felt that their determined and outgoing personality had a key role in recovery. The identified attributions of recovery processes correspond to protective factors in the models of mental health protection, resilience factors in models of stress and trauma resilience, and key principles for interventions in the aftermath of massive trauma. However, specific factors were identified in the present study that have not been reported before. These are in particular a strong orientation towards the future, reciprocity in receiving and giving social support, meaningful activities that ensure social recognition, and contextual aspects of provisions of mental health treatment. These findings can inform psychosocial interventions for survivors of trauma.
